# Eating experiences and quality of life in patients with larynx cancer in Spain. A qualitative study

**DOI:** 10.1080/17482631.2021.1967262

**Published:** 2021-08-25

**Authors:** Carmen Cipriano-Crespo, David Conde-Caballero, Borja Rivero Jiménez, Lorenzo Mariano-Juárez

**Affiliations:** a Faculty of Health Sciences, University of Castilla La Mancha, Talavera de la Reina, Spain; b Faculty of Nursing and Occupational Therapy, University of Extremadura, Cáceres, Spain; c Polytechnic School, University of Extremadura, Cáceres, Spain

**Keywords:** Cancer, eating, qualitative, narrative-based-medicine, experience

## Abstract

**Purpose:**

This paper aims at describing the eating experience of people diagnosed with and treated for laryngeal cancer. Going beyond the mere conceptualization of “after-effect” or the quantification of the disease’s impact on the basis of standardized questionnaires, we present a qualitative analysis of the narratives of such experiences.

**Methods:**

Ethnographic study. Data is obtained from conversations, semi-structured interviews, participant observation, and written documents. A discourse analysis of the narrative information was conducted, with process coding and using the constant comparative method, inductive content analysis, category analysis, units of meaning associated with each other, and triangulation.

**Results:**

The impact of cancer on eating processes is not limited to nutrition, but also affects the social and cultural value of food, which is put into question. The symbolic and social values which accompany the traditional way of eating are modified, which is connected with impaired quality of life.

**Conclusions:**

The impact on the eating process and its relationship with quality-of-life impairment are clear and connect with the importance of eating ways in culture and social organization. Greater attention should be paid to these contexts in clinical practice, which can affect even more than the impact on communicative processes.

## Introduction

Laryngeal cancers represent one-third of all head and neck cancers and can be a significant source of morbidity and mortality. Smoking is the most significant risk factor for cancers of the larynx, associated with an estimated 70% to 95% of all cases (Koroulakis & Agarwal, 2020). A total of 277,394 cases are diagnosed in Spain each year (Sociedad Española de Oncología Médica, 2020). Until recently, its treatment was exclusively surgical, and although nowadays efforts are made to preserve the organ (Bonomi et al., 2018), in many cases the impact on communicative (Fung et al., 2005) and eating processes is inevitable. In those cases, the patient’s voice is lost or changed, and there is total or partial ageusia or anosmia. The disease bursts into the patient’s life fiercely. The impact on eating processes includes the erosion of the emotional and social sphere in a way which is rarely considered in clinical records. Once the nutritional content is ensured, there is a tendency towards the invisibilization of this entire world of emotional and social experience of food (Cipriano Crespo, 2018).

Anthropology studies how people with disabilities function in the different cultures and contexts in which they are immersed, to understand what their lives are like and to improve the quality of their lives. It is important to study it within the culture to which they belong, and to consider the way they share beliefs, values, and expectations, otherwise it would be difficult to understand. To this end, they promote mutual engagement and collaboration in research and training by anthropologists (Kasnitz & Shuttleworth, 2001). Therefore, anthropology aims to situate the disability caused by the presence of laryngeal cancer as a social and cultural phenomenon and to describe its variability of meanings according to different contexts, focusing on the person with a disability as a unique being and the way of coping with the situation he or she is experiencing.

The link between cancer and food has been extensively referenced. Most of these describe aetiological relationships between different consumption choices or preferences (Chacón-Cuberos et al., 2018; Emenaker & Vargas, 2018) or the analyses of the preventive values associated with certain diets. For example, it has been pointed out that between 30% and 40% of all cancers could be prevented with an appropriate diet (Donaldson, 2004; Mentella et al., 2019; Salvatore Benito et al., 2019). Other approaches have focused on the impact of cancer and/or its treatment on the patients’ nutritional state (Fuchs et al., 2008; Gangadharan et al., 2017; Ravasco, 2019). The impact of the disease on eating processes. Concern also for the problems caused by the different treatments such as nausea, loss of appetite, dysphagia,^1^ anosmia,^2^ ageusia.^3^ However, the approaches centred around the impact of the pathological process on social and cultural dietary patterns and the correlation on people’s quality of life are much more exiguous. A large part of these approaches has been grouped around the category “quality of life”, even though the reflection on the eating process has always had a secondary status as opposed to the communicative impact (Karlsson et al., 2015; Tuomi & Karlsson, 2020). Some papers have highlighted the impact on dietary patterns (Jochems et al., 2018; Steck & Murphy, 2020) but the phenomenological analyses of such changes in this group’s daily life are scarce. The alteration of food practices should be a cause of concern not only from a nutritional point of view but, since these are understood as central aspect of sociability and occur in specific cultural contexts, they have an impact on the self-perception of quality of life and also on one’s own identity (Ottosson et al., 2013).

The need to gather the narratives and experiences of cancer patients and their families begin to be stressed (Garassini, 2015). As well as to gather the emotional and social experiences of cancer patients who live in care, nursing homes (Kristensen et al., 2019). The interest in these areas of experience is clearly evidentialist according to the theses of Narrative-Based Medicine (Charon, 2001; Greenhalgh, 1999; Greenhalgh & Hurwitz, 1999; Hurwitz & Charon, 2013). There is few evidence more revealing than the patients’ suffering itself, narrated in personal terms. The need to gather this type of evidence is therefore assumed, evidence traditionally forgotten since they capture the subjective part of patients, which is not included in the medical history (Greenhalgh & Hurwitz, 1999; Mariano Juárez & Cipriano- Crespo, 2012). If, as over a century of social research has emphasized, eating is much more than nourishing the body (Contreras, 1995; Espeitx & Gracia Arnaiz, 2012; De Garine & De Garine, 1998; López García, 2012), we need to know the impact of the disease on these social and cultural dimensions of the eating process. As regards the eating process, we need to evidence what the disease destroys which goes beyond the biologicist approach of macronutrients count.

## Methods

This is a qualitative study, of an ethnographic nature. Data collection and analysis followed an inductive approach (Einarsson et al., 2019; San Martín Cantero, 2014). Categories were established following the analysis of raw empirical material, using the constant comparison method within the theoretical framework defined, and following the contributions of the grounded theory (Bovio et al., 2008; Corbin & Strauss, 2008). This allowed for a better analytical understanding of the experiences (illnesses) with feeding difficulties (Larsson et al., 2003; Strauss & Corbin, 2016). This theory studies social phenomena in natural contexts, such as pain experiences in people hospitalized with cancer and is also used to study the quality of life of people suffering from laryngeal cancer as it is intended to do in this text (Vivar et al., 2010).

### Participants

The final sample included 15 participants (13 male and 2 female), patients in different stages of disease, within an age range from 54 to 77 years, and a mean age of 63.8 (Table I). In all of them, their eating habits have been modified by the disease or the treatment. All live in the region of Castilla-La Mancha (Spain). The participants of the study were contacted through access to support groups for cancer survivors.

**Table I.** Characteristics sample of research

Age	Sex	Start of symptons	Diagnosis	Problems
55	Male	March 2013, diagnosis in January 2014	Squamous cell carcinoma of the pyriform sinus	Difficulty eating, swallowing fear
60	Male	Symptoms began in December 2007, diagnosis in 2008	Laryngeal cancer	Loss of pleasure, self-imposed social isolation
60	Male	Symptoms began in September 2014	Laryngeal cancer	Problems preparing food
55	Male	Symptoms began in February 2014, diagnosis in July 2014	Laryngeal cancer	Undervalue: feels worth less than others
66	Male	Diagnosis in January 2013	Laryngeal cancer, total laryngectomy	Loss of pleasure, eating alone
59	Male	Diagnosis in May 2012	Laryngeal cancer	Shame for eating slowly and taking other types of food that are not considered normal
54	Male	Symptoms began in 2013, diagnosis in September 2014	Laryngeal cancer	Nostalgia
71	Male	Diagnosis in September 2011	Laryngeal cancer	Anosmia, loss of pleasure in eating due to loss of sense of smell
65	Male	Began 2009	Laryngeal cancer	Eating alone
77	Male	Began in November 2011	Laryngeal cancer	Giving up eating at restaurants
75	Male	Symptoms began 2004	Laryngeal cancer	Sadness about tube-feeding and not enjoying food
60	Male	Diagnosis in 2014	Laryngeal cancer	Ageusia
70	Female	Symptons began 2009	Laryngeal cancer	Loss of pleasure with food
60	Female	Diagnosis in 2015	Laryngeal cancer	Fear to feed for the presence of dysphagia
70	Male	Symptoms began 2009	Laryngeal cancer	Sadness about tube-feeding and not enjoying food

The inclusion criteria were: a) being at least 18 years old, age of majority in Spain, b) having a functional deficit in the occupational area of eating caused by cancer, and c) signing the informed consent form, thus giving permission to record the interview. Those with cognitive impairments, those who did not give consent, those who did not have a functional deficit on the occupational area, and those who refused to be audio-recorded were excluded from the study.

### Data collection

Data collection was based on in-depth interviews, guided by a series of semi-structured questions (Table II) which allowed for the exploration of new themes emerging during the course of the interviews (Carpenter, C.; Suto, M. *Qualitative Research for Occupational and Physical Therapists: A Practical Guide*; Wiley-Blackwell: Hoboken, NJ, USA, 2008; p. 202. ISBN 9781405144353) Neumann & Neumann, 2018, Emans, 2019). All the interviews were audio recorded.

**Table II.** Guide of themes of semi-structured interview

Aim/lines of inquiry	Questions
Onset of the disease	*When did it all begin? Therapeutic pathway*
Forms of food preparation	*How do you cook food? What differences are there from before cancer?*
Ways of eating	*Tell me about a normal meal. Do you have any difficulties eating?*
Acceptance of change	*Tell me about the first few days*
Presence or absence of pleasure in eating	*Did you enjoy eating before the disease?; and now?; do you eat the same food as before the disease was diagnosed?*
Views on food	*What is your day-to-day food like?*
Emotional aspects	*How do you feel when you are preparing meals? And when eating? What are your feelings about food?*
Discourses about the body	*What changes has cancer caused in you? Is there something different now? Can you give an example?*
Eating sociability	*What are celebrations like in your home? Can you tell us about your outings in restaurants?*

The interviews were conducted in the participants’ homes, or in occupational centres. Empirical material based on observation were also collected from places such as kitchens, eating areas, storage centres, pantries, or even catering public areas. Some meals were shared with them in these spaces and observation of the speech therapy room where they worked on feeding was also carried out. Participants were contacted through the support groups for cancer survivors. One of the investigators conducted the interviews, which concluded when it was considered that discourse saturation was reached. Two of the investigators carried out independent analyses which were put in common. The script of the semi-structured interview was organized with categories from the literature on cultural aspects of the eating process, open to emerging themes which were not in the initial script. The interviews lasted from 42 to 91 minutes, with an average duration of 62 minutes.

### Data analysis

The analysis and interpretation of the data was done based on the Grounded Theory (Strauss & Corbin, 1994) The overall transcription of the interviews was carried out with a criterion of strictly literalism which reflected the personal style of each interlocutor (Pujadas, 2000). Categories were identified and analysed through systematic content-coding: 1) Preliminary units of analysis were established after a first reading of the transcripts; 2) Using the constant comparison method, open-coded data were grouped into concepts, and categories generated accordingly. Initially, 37 categories were defined, which were later grouped into 5 categories. Once data had been broken down, relationships between categories and subcategories were actively and systematically analysed in a process of axial coding (Bonilla-García & López-Suárez, 2016). Finally, following a process of elective coding, categories were grouped into 5 major themes.

### Ethical considerations

This study was approved by the Clinical Research Ethical Committee of the Talavera de la Reina Integrated Management Area (CEIm del AGI of Talavera de la Reina in Spain, Hospital Nuestra Señora del Prado, ref: 18/2014). The research was in line with the ethical principles outlined in the Declaration of Helsinki and the Belmont Report. The data processing was subject to the strictest rules about the ethical implications of research. All the data were treated confidentially and have been protected by the Spanish Law 15/1999, of 13 December, of Personal Data Protection. The gathering of information about the protagonists of the research has been voluntary, with their prior written informed consent. To ensure confidentiality, the names that appear in the study are fictitious.

## Results

**Figure 1.**
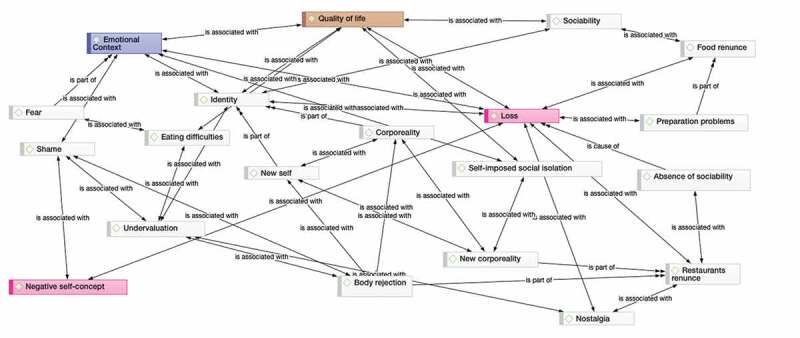
Network of relationships between categories

The accounts of eating experiences are described in terms of loss where the experience of eating appear as a reference of a past before the illness that constructs the difference with respect to a present where many of the pleasures derived from eating plot have been lost. The concept of after-effect refers to that decrease in functionality, which is considered a change for the worse. The plot of the stories is articulated through nostalgia: it is accepted as an inevitable part of survival, and a way of eating is described that refers to a less valid life. In Table III we describe the 5 major themes that emerge from the analysis. Figure 1, created with the software ATLAS.ti, relates the different categories and major themes worked on in the study.

**Table III.** Analysis of categories and themes

Themes	Subcategories	Verbatim
1. Quality of life	1a. Difficulty eating	*Food clumps up in my mouth, like children say*
	1b. Giving up some food	*I eat alone because I know I must*
	1c. Loss of pleasure	*No smell, no taste, I don’t do it any more* *“you eat hospital food in your own home”*
	1d. Problems preparing food	*It felt like I was eating tin instead of food*
2. Emotional aspects	2a. Fear	*I was afraid of choking*
	2b. Nostalgia	*The smells of food that took me back to childhood, to granny’s meal*
	2c. Shame	*I am embarrassed that food spills off the sides*
3. Identity	3a. New self	“you have the feeling that you are not the same person”
	3b. Low self-concept	“I think of the things I can´t eat any more and I feel as if I was damaged”.
4. Corporality	4a. Alien corporality	*“it is not me who is eating, a machine is” “eat through the rubber tubes”* *“it’s like having a mouth that is not my own, another … ”*
	4b. Undervalue	*I looked in the mirror and I was afraid to see myself*
	4c. Rejection	*I feel people don’t want to be with me*
5. Sociability	5a. Eating alone	*Eventually after feeling bad many times I don’t go any more*
	5b. Self-imposed social isolation	*I prefer to eat apart so that they don’t see me*
	5c. Giving up eating out	*I used to come to celebrate, but now going out means to face nothing but problems*

### Quality of life

The category “quality of life” has been intensely addressed in a quantitative manner, from questionnaires such as EuroQol-5D or the quality of life scale WHOQOL-BREF (Lin et al., 2019; Malm et al., 2016; Pedersen et al., 2019). However, the complexity of its definition, open to the variability of experience, tends to overshadow spaces and meanings that build and shape it. In this study, the concept is defined inductively, incorporating the various phenomenological areas which are related to the eating process: the emotional context, the sociability, the acceptance of a new corporality, and the impact on the definition of a “lessened” identity.

The deterioration in the eating process encompasses perceptions about the artificiality of the latter (purchasing food in the pharmacy instead of the supermarket, having jars that seem medicines in the pantry, or the eating process itself aided by support products, “eat through the rubber tubes”. The entire culinary universe is altered temporarily or permanently and is lived as a loss in quality of life. Coping strategies include attitudes of “rejection” or attempts to normalize and go back. Paloma refuses to undergo a gastrostomy because she knows that, if she consents, her quality of life will be affected:

(…) even if it takes me an hour to eat I have to do it myself, eating through the rubber tubes is not the same as seating and chewing food. As long as I’m able to chew I’m going to do it, if I die at least I die without the tubes.

Eating certain foods is sometimes life threatening. The choking hazard is there, which shapes the scope of what is edible on the basis of giving up some foods and preparations. Nostalgia and what cannot be eaten make up a feeling of unease which forms that decline in quality of life. Food, once a place for pleasure, turns into a necessary routine: “*I eat only because I know I have to eat to get well, but I don’t enjoy what I take*” told Ángel.

Patients identify the loss of smell as one of the alterations that makes a good meal more difficult for them, because, without it, they cannot evoke memories of the past. Without smell, it is more difficult to feel pleasure with food. Antonio began to feel that food tasted “*like metal*” shortly after starting the radiation sessions. He made strenuous efforts because “*it felt like I was eating tin instead of food*”; everything tasted the same and, for him, eating was an obligation and not an enjoyment. Fernando does not drink water and struggles between thirst and disgust, as a result of dysphagia. The disease eventually defeats personal taste and patients experience a life in which they even find it hard to quench their thirst:

I miss the feeling of quenching my thirst with water, because I used to drink more than one litre a day, and with the juices I drink less because they don’t quench my thirst, I don’t like them, but I’m more disgusted by the thickener when I drink water.

A desire shared by most of the participants is to recover the old way of eating, which provided pleasure and help them feel in control of their own lives. Jesús is fed by gastrostomy, something very far from his previous eating. His meals now are “*more artificial*”, it is purchased only in pharmacies, almost a medicine, and it does not allow him to recover the sense of feeling fed:

(…) it doesn’t feel like I’m eating because I can’t smell or taste what gets into my body. Putting food in my mouth, smell it and taste it, that’s the way I like to eat. I enjoyed that and many happy moments came to my mind about situations lived with food, now with this way of eating I can’t do that

These are other forms of eating which allow nourishing the body, but do not help to satisfy the desire, the enjoyment when putting appetizing food in one’s mouth, to “*have a real meal*”, in the words of the participants. Ramón does not forget the pleas he made to his wife to give him “*real food*”.

The ways of cooking food change due to the consequences of cancer. Boiling closes off other less appetizing, but more easily digestible, forms. These ways of eating entail a necessary adjustment in meal preparation. Now it is necessary to crumble up food in tiny bits which avoid or reduce the risk of choking. An example of this is what happened to Luis, who needed to break the noodles before boiling:

(…) that was very difficult because breaking something so thin in smaller pieces is really complicated, but if I didn’t do that, it hurt to swallow, as if they stabbed my throat

The changes caused by cancer do not only affect the new meals that they need to have, or their preparation, they also affect the utensils used for them. Those affected by cancer have to include new cooking utensils in their kitchens. Jars of prepared food, syringes, and feed pumps will be part of the new daily life of cancer survivors. The impact of that is lived as a loss of quality of life, which is also present in the accounts about emotions.

### Emotional aspects

Quality of life is also built on a particular emotional context. In this group, the emotional tone is recreated negatively on the basis of nostalgia, loss, and helplessness. The losses caused by cancer are lived with longing and melancholy, and the aim is to readapt the new sensations obtained with food. As well as fear.

The accounts about fear of not knowing how to eat, fear of choking, are lived from suffering. There is nothing attractive or pleasant in eating artificially. Some of the participants who live this situation eat on the couch, *plugged in* to a machine, losing their taste for food, and feeling a growing fear of the new ways of eating:

You are really scared that food gets stuck in your throat and you can’t breathe, with the added risk of having a constant open wound that can get infected. (Jesús)

When one eats with fear, food becomes a torture, a mechanical act therefore defined as an obligation and a burden. The fear of eating provokes anxiety and anguish, feelings which hinder a normalized life, reducing the list of edible meals. You do not eat or drink what you want, but what you can. Sometimes fear increases due to the obligation imposed by those who make them eat, as in the case of Javier:

(…) I found it very hard to swallow and eat with the tube, it was a little unpleasant and I was afraid of choking, they didn’t explain anything, they only forced me to do it and that’s it.

The accounts describe the acceptance of the clinical obligation to eat that way, but highlight complaints about not being understood given the abyss that opens up with these new ways of eating. The lack of emotion provoked by food is recreated on the basis of nostalgia, always defining the present as lessened, as “lacking”: *“I miss the smells of meals that took me back to childhood, to the granny’s meals”* as Mateo said. The emotional biography anchored in the food memory can only create comparisons of a change for the worse. And there is also envy. Envy towards those who can smell and taste the food that they will not be able to enjoy any more, even family and friends:

(…) Not smelling or tasting food was as if I wasn’t eating at all, I only performed the act of chewing and swallowing, nothing else, without enjoying what I was doing, and I feel envious when other people can do it. (Ramón)

Another emotion that the participants feel is shame. When they depend on others for eating and other activities of daily living, they feel that they are undermining their dignity with the way they treat them, and this is often lived as a process of infantilization:

(…) I was scolded for everything, because I ate too slowly, because I didn’t eat, and eventually they had to feed me. They did it too fast and it made me feel sick, and I felt like a child who is scolded for everything. (Benito)

That emotional universe recreated on the basis of loss is explained by the social and cultural value of food. When eating habits are modified, a negative emotional universe is created, which in addition affects one’s own identity directly.

### “Damaged” corporality

A large part of the discourse is focused on the reflection about a body which is experienced as other, as defective. A body that has been patched up, but which is not the same as before. The mouth is described as diminished, unable, not only to speak normally, but also to eat—socially- as it should. This type of cancer provokes substantial changes in the individual which significantly affect external appearance, which leave a mark on the persons who suffer it: having a nasogastric tube, having a stoma in the throat. Mouth and throat have turned into body parts that cannot be trusted: they can fail to perceive flavours, to swallow food. In some participants this body parts disappear and turn into a stoma or a tube connected to a machine. This new mouth enables nutritional needs to be met, but does not satisfy the social or cultural demands. Life sinks through this new mouth, the reconstruction of a new body is necessary:

(…) It’s true that what goes down the tubes staves off my hunger, but it is not me who eats, a machine is doing it for me, and to me that’s not eating. (Jesús)

Cancer has an effect on a bodily experience of discovery under feelings of mistrust and sadness. Remarks about meal times highlight the concentration required to prevent choking, eating slowly, away from distractions, from conversations, and from noises from the TV, only the individual and the dish. What used to be an unnoticed routine entails now exhaustion and attention.

The participants who have to eat using an enteral feeding pump feel that this keeps them away from those with whom they used to share meals, and reminds them of the hospital environments where they have spent some time:

(…) my belly is what keeps me alive, it’s where I’m plugged in to the machine I have and where I get the food they buy for me in the pharmacy. It goes down drop by drop, sometimes if it doesn’t pass through properly it gets stuck and food doesn’t get in. It’s a routine that can be boring, but necessary, unfortunately I can’t eat otherwise. (Jesús)

I feel that people reject me when they see the tube in my nose, that’s why I want the tubes in my stomach, so that nobody will frown at me because nobody will know that something is wrong with me. (Jesús Javier)

These body changes alter the person’s appearance, causing the already mentioned emotional universe, but also self-confinement practices which have an impact on personal and food sociability.

### Identity

The changes perceived in the body and the new ways of eating are lived as a move away from the pre-cancer *food normality*, which includes modifications in the very concept of identity. Cancer patients with tracheotomy can see themselves with the damaged image that the *other* projects to them, which causes personal harm and makes them feel “weird”, different to the others.

A three-year-old neighbour of mine doesn’t want to talk to me since I had surgery because she gets scared of the sound coming out of my body, that seems to come from a cave, and it makes me sad. (Rafa)

The participants complain of difficulties to accept that change, which influences directly the perception of self-identity. Of course, these self-identifications do not result only from body modifications, but above all, from the new impositions in eating. Sometimes they depend on others to buy and prepare food, or to help them eat. Meal times are different, slower. And with that, the identifications of a less valid, less capable self appear. Benito described it as follows:

The fact that my girls had to feed me was a shame for me, I had to keep on protecting them and not the other way round.

### Sociability

This impact is related to the social sphere linked to food. In Spain and in other contexts, it is impossible to separate eating from social interaction. The effect on quality of life also involves giving up that social exchange, confining oneself in some spaces and hours of the home, not going to restaurants, and avoiding parties and celebrations. The accounts highlight the importance of the stares, the control over meal times, the tools that “make them different”. “*It is a disease that separates you from society*”, said Jesús. If eating is mainly a social act, the new ways seem to lead them to “eat alone”, or at best, to eat with equals:

(…) eventually after feeling bad many times I don’t go anymore. I only feel good at the lunches of the association, I don’t care if any of my comrades tells me to clean myself because food is spilling off, he has gone through the same and I don’t have anything to explain away. (Fernando)

At first I ate with the rest, but food spills off all the time and one day I heard a man saying aloud that it was disgusting, and he turned his back on me so that he didn’t have to see me. Since that day I’d rather eat alone. (Paloma)

Mealtime ways include conceptions of times, paces or customs which are modified by cancer. The accounts describe here a process of giving up group meals, thus preventing others from hearing noises, seeing mucus, or having to wait. Although this is common in the descriptions of outings in restaurants, it also happens at home: they choose isolated places, or even different hours. And what remains, again, is absence.

If social everyday life tends to be avoided, the sense of loss is greater with celebrations. Many participants talk about avoiding any celebration, which again has an impact on that process of social isolation. If food is a major vehicle for celebration, the new way of being in the world means shying away from that party.

in some meals with friends, I had to get up from the table and go to the toilet to spit out the piece of food that had got stuck, throw it up, or swallow it. At the beginning I used to get very nervous because I thought I was going to throw it up on the table. That is why I try not to eat out. (Luis)

With my condition we don’t celebrate anything anymore … only my children’s birthdays … we don’t celebrate anything because they all know that I have a bad time because I can’t eat like the rest, and to spare me the suffering we don’t celebrate anything with meals, that used to be the way we celebrated everything at home … well … as in almost all homes, we are in Spain, and here we eat well and drink well. (Jesús)

## Discussion

The study group show problems in the eating process after the onset and treatment of cancer, even years after its cure. A large number of previous studies underline this very issue (Bovio et al., 2008; Einarsson et al., 2019; Kristensen et al., 2019), but the focus has always been on nutrition, rather than on the problems derived from the symbolic value of food practices. There are major changes and implications in the protagonists’ daily lives (Hopkinson, 2016; Larsson et al., 2003; Rossi et al., 2014) which affect the sense of competence and self-esteem. But, above all, these changes have a direct impact on the quality of life of these persons, who see how it declines in different areas centred around the act of eating in this “new way”. Some previous research (Hopkinson, 2016; Kristensen et al., 2019; Patterson et al., 2015) has also highlighted the experiences of loss, in some cases irreversible, which causes feelings that range from rejection, to resignation, to subsequent struggle and acceptance of this new normality that they happen to be living.

In this research there have been constant references to the difficulty in dealing with physical changes, which are harder because the patient is aware of their permanent nature. As other studies have already pointed out, the loss of smell has far-reaching implications that bring about problems of safety, hygiene, and a limited response to pleasant smells (Steyn & Green, 2018). These limitations have a direct impact on how the patient faces the act of eating, and how there is a loss of pleasure and, in a way, of meaning. Our participants, as those of the study by Ottosson (2013), described how the pleasure of food had disappear and had turned into something imposed, and how some participants ate only because they had to. Meals become one more day-to-day obligation, as in other studies with cancer patients (Kristensen et al., 2019; Ottosson et al., 2013).

The clinical literature has nevertheless paid secondary attention to the impact of the new way of eating on the basis of available evidence about the social senses and meanings given to food (Contreras, 1995; De Garine & De Garine, 1998; López García et al., 2016). The notion of “loss” present in the experiential accounts is in line with all the literature that the social sciences have produced about the symbolic importance of eating practices. In a context such as Spain, where eating is about macronutrients as much as about social relations, the disease bursts in affecting social spaces such as after-meal talks or views about pleasure, with a secondary impact on identity and quality of life. The preparation of meals is also a metaphor of care, which allows finding psychological and emotional well-being with those with whom one shares it (Kaplan, 2000). Cooking means as well to have the capacity to satisfy other people’s need of getting fed, and the capacity of providing enjoyment and pleasure with food (Criado, 2004; Kostyra et al., 2017; Velasco et al., 2012); this capacity is weakened in our participants, which leads them to a lessened self-worth.

The methodology of using in-depth interviews, the active listening of the patients’ discourses, makes it possible to address sensitive personal issues. In those we can find the presence of fear. The fear of eating due to dysphagia also appears in other studies conducted on persons with head and neck cancer (Patterson et al., 2015; Van Roij et al., 2019), although here we show how it goes far beyond: it seeps into social practices, fear of being seen, of being infantilized, of being stared at.

It has been pointed out how modifications in the mouth and associated voice problems have a direct impact on the quality of life of larynx cancer patient (Etter et al., 2013). But the mouth is also a vehicle for the social exchange that takes place through food, an aspect which is barely dealt with. Although feelings of shame and embarrassment have been described in works such as Kristensenn’s (2019), the methodology used enables other feelings like envy to emerge. As regards corporal identity, some studies show that patients with a tracheal stoma after a total laryngectomy, or with extensive scars in neck and face, usually suffer depression and loss of self-esteem, which affects their quality of life (Babin, 2002; Nahum & Golden, 1963). There are accounts about feeling ugly because of the use of catheters, like those provided by Guimaraes (Guimarães, 2012) where, despite being acknowledged as necessary in most cases, it is perceived as something negative that breaks one’s normal appearance. Many patients suffer a feeling of abandonment, betrayal and deprivation of identity induced by cancer, factors which promote individual isolation, marginalization or even exclusion, thus inevitably reducing their perceived quality of life (Babin et al., 2005, 2008). We could point out a quite direct connection between the world and the space of identity, and how it is related to the social sphere and its direct influence on quality of life.

The literature has described a reduction and/or loss of social relationships of larynx cancer survivors (Kristensen et al., 2019; Larsson et al., 2003), something that can also be found in the participants of this study. Although the usual approach has emphasized the problems derived from altered communication, this study supports the idea of the importance of “food ghettoization”, that is, the process of self-confinement described above.

The limitations of the study stem from the methodology used. We do not know whether the importance given to the eating process is the same in other social and cultural contexts, so comparative analyses should therefore be conducted. Without a quantitative assessment of quality of life, it is impossible to establish comparative relationships with the inductive methodology on which the concept is built here.

## Conclusion and applications of the results

The new ways of eating can provide the nutrients that the body needs, but they do so at the expense of a social and cultural anorexia: the new ways of eating drive away the spaces of pleasure and sociability, producing negative emotions which cause permanent changes in self-identity. The social and cultural gap created in loss defines a scenario where quality of life is challenged. Further analysis is needed from these approaches which highlight the value that the social sciences have placed on food practices. Only then will we be able to generate biopsychosocial care and attention practices.

The social and cultural gap created by the loss of food defines a scenario in which the quality of life is clearly diminished. It is necessary to deepen the analysis of qualitative approaches that highlight the value of food practices from other aspects and positions of the social sciences. This is the only way to generate care practices that include the different social and cultural aspects.

The subjectivities of food and life histories in the medical records of patients with laryngeal cancer need to be incorporated into the healthcare setting. Considering the feelings, they have about the change that cancer has brought about in their lives and those of their families can make patients more involved in the treatment offered to them. This should go beyond the provision of micro- and macronutrients and include psychological and social spaces and processes of interaction with definitions of personhood. Health professionals involved in the therapeutic process should place patients’ narratives of distress at the centre of their practice, considering them as evidence of the highest order.

## Notes

1. Dysphagia:Difficulty or absolute impossibility to swallow solid food and liquids.

2. Anosmia: Loss of smell.

3. Ageusia: Loss of ability to taste food.
